# Regional differences of physical fitness and overweight and obesity prevalence among college students before and after COVID-19 pandemic since the “double first-class” initiative in China

**DOI:** 10.3389/fpubh.2023.1252270

**Published:** 2024-01-05

**Authors:** Qing Jiang, Xin Huang, Zuoliang Wang, Xinghong Dai, Rongxuan Li, Di Cui

**Affiliations:** ^1^School of Physical Education, Hunan University, Changsha, Hunan, China; ^2^Hunan Students’ Physical Fitness Test Data Management Center, Changsha, Hunan, China

**Keywords:** COVID-19 pandemic, physical fitness, overweight and obesity, DFC, college students, regional differences

## Abstract

**Introduction:**

Physical fitness has been widely recognized as a powerful marker of health in children and adolescents, and it negatively affected by the COVID-19 pandemic. The construction of world-class universities and first-class disciplines, known as the “Double First-Class” Initiative (DFC), is a major commitment made by the Chinese government to adapt to changes in the educational environment, both domestically and internationally, in order to promote the development and practice of international higher education. The aim of the study was to look deep into the regional differences of physical fitness and overweight and obesity prevalence among college students before and after the COVID-19 pandemic since the DFC.

**Methods:**

The original physical fitness parameters of students from 10 DFC universities and colleges in Central South China were downloaded from the official website of Chinese National Student Physical Fitness Database (CNSPFD) and then divided into 3 groups based on the pandemic periods: pre-pandemic (2019), the first year after pandemic outbreak (2020), and the second year after pandemic outbreak (2021). All the data were stored in Excel 2010, analyzed by SPSS 17.0, and plotted with ArcGIS 10.4.

**Results:**

The total “fail” percentage (from 9.19% in 2019 to 12.94% in 2021) and the prevalence of overweight and obesity in boys (from 22.53 to 29.25% in 2021) exhibited a continuous increase year by year, and among all the physical fitness indicators the score of strength in boys and endurance quality in all individuals were the lowest in overweight and obesity groups. Students with ‘fail’ rate developed from northern and northeastern province to southern areas from 2019 to 2021. For grade 2019th, overweight and obesity students who also failed the test had covered nationwide and the most affected areas including northeast, east, as well as central north in senior year. The distribution of overall fitness assessments in Hubei province was in accordance with the national data, and the overall scoring growths in both class of 2021st and 2022nd were measured with a negative increase (*p* < 0.01).

**Conclusion:**

The government and related functional departments should take into consideration the student regional sources, especially in western and northeast regions of China, and school polices and physical education (PE) teachers should pay more attention to put training efforts on endurance for all adolescents and strength for boys and the group of overweight and obesity who also failed in the standard test, when designing specific interventions to promote physical health and counteract the negative effects of COVID-19 pandemic in college students.

## Introduction

Physical fitness, which refers to the ability of our body systems to work together efficiently, maintain health and perform daily activities, has been widely recognized as a powerful hallmark of health among children and adolescents, and the level of individual physical fitness is positively associated with multiple health benefits (1). Previous studies showed that individuals with a high level of physical fitness typically have a lower risk of chronic diseases and premature death, including cardiovascular disease (2), cancer (3), hypertension (4), and mental disorder (5). Regular physical fitness measurements and evaluations have emerged as state strategies to monitor the health status and trends of children and adolescents worldwide (6–15). In China, the National Student Physical Health Standard (revised 2014, short for standard below) has been launched by the Ministry of Education since 2002 (16), collecting and monitoring the physical fitness information of children and adolescents at school age.

Overweight and obesity among the younger population are predominately a result of a sustained positive energy balance, stemming from a combination of excess dietary energy intake (mainly due to poor eating habits) and reduced energy expenditure (due to lack of physical activity and prolonged sedentary activities) (17, 18). In March 2020, the World Health Organization (WHO) announced the coronavirus disease 2019 (COVID-19) pandemic (19). Up to December 2022, over 645 million confirmed cases and over 6.6 million deaths have been reported globally since COVID-19 was firstly identified in Wuhan, China, in December 2019 (20).The governments of most countries enacted numerous restrictions on movement and interactions, including quarantine, lockdown, and community containment, to control the spread of COVID-19. In China, all schools and universities were closed resulting in students being quarantined at home for at least 6 months (from mid-January 2020 to mid-June 2020). There are studies conducted worldwide focusing on the effects of pandemic restrictions on physical fitness in children and adolescents, and the overwhelming majority of results have shown, that physical fitness of children and adolescents has been negatively affected by the pandemic constraints due to reduced physical activity and increased sedentary behaviors (21–29). However, little research has been done to explore the impact of COVID-19 crisis on the physical fitness of college students especially among overweight and obesity groups and students from the epicenter of the outbreak, therefore, the present study aims to delve deeper into the regional differences of physical fitness and overweight prevalence among college students in a longitudinal way, peculiarly before and after COVID-19 pandemic. In September 2017, the Chinese Ministry of Education, Ministry of Finance, and National Development and Reform Commission jointly released the detailed lists of universities and disciplines to be developed under the “Double First-Class” (DFC) initiative, promoting the global recognition of native higher education system by 2049. The physical health status of students is closely related to the level of higher education, so the candidate universities and colleges recruited in the present study are randomly and variably ranked on the DFC list.

Based on the above, this study analyzed the overall and individual physical fitness parameter evaluation of students for three consecutive years from 2019 to 2021 and further explored regional differences with the aim of providing references to build a precise state strategies and interventions to counteract the negative effects of COVID-19 pandemic on physical fitness and promote physical fitness among college students.

## Methods

### Study design

A retrospective study was used to track the physical fitness data of college students from 2019 to 2021, and evaluate the pandemic impacts on physical fitness indicators and regional distribution features. The original physical fitness parameters of 684,227 students from 10 DFC universities and colleges (represented by A-J) in Hunan province were downloaded from the official website of Chinese National Student Physical Fitness Database (CNSPFD). By excluding incomplete or missing data in surveyed years, a total amount of 103,072 subjects (50,696 boys, and 52,376 girls) were screened, and the sex ratio was close to 1:1 (Table 1). The data were then divided into 3 groups based on the pandemic periods: pre-pandemic (2019), the first year after pandemic outbreak (2020), and the second year after pandemic outbreak (2021).

**Table 1:** Number and distribution of participants by school and gender.

School	Total(N)	Boys		Girls	
		N	%	N	%
A	15,765	9,740	61.78	6,025	38.22
B	9,071	4,936	54.42	4,135	45.58
C	8,354	3,071	36.76	5,283	63.24
D	12,260	6,642	54.18	5,618	45.82
E	9,327	2,555	27.39	6,772	72.61
F	6,522	2,173	33.32	4,349	66.68
G	7,959	3,480	43.72	4,479	56.28
H	8,485	4,507	53.12	3,978	46.88
I	11,761	5,612	47.72	6,149	52.28
J	13,568	7,980	58.81	5,588	41.19
Total	103,072	50,696	49.19	52,376	50.81

### Parameters

The *Standard* has been considered the largest nationally representative survey of the health status in China among children and adolescents including seven indicators, BMI (body mass index, a person’s weight in kilograms divided by the square of height in meters), vital capacity, 50-m-run, long-jump, sit-reach, 800-m-run for girls/1000-m-run for boys, and 1-min-sit-up for girls/pull-up for boys, and all these parameters were utilized to assess students’ body composition, cardio-pulmonary function, agility, explosiveness, flexibility, endurance, and strength individually or in combination. The overall physical fitness was scored by the formula defined in the *Standard* through weighting BMI by 15%, vital capacity by 15%, 50-m-run by 20%, long-jump by 10%, sit-reach by 10%, 800-m-run/1000-m-run by 20%, and 1-min-sit-up/pull-up by 10%. According to testing-score, all the assessments and the overall evaluation were classified into four level: ‘excellent’ (scored 90.0 and above), ‘good’ (scored 80.0–89.9), ‘pass’ (scored 60.0–79.9), and ‘fail’ (scored below 60.0). Notably, the *standard* defined the body composition using the cut-off points: an individual with a BMI between 24.0 and 27.9.0 was considered ‘overweight’ (scored 80); a BMI greater than 28.0 is defined as ‘obese’ (scored 60).

### Measures

#### BMI

All participants were required to be barefoot and wear light clothes while measuring body height and weight. Both body height and weight were measured using a portable stadiometer. Body height was measured to the nearest 0.1 cm and body weight was measured to the nearest 0.1 kg. BMI was calculated as the body weight in kilograms divided by the square of the body height in meters (kg/m^2^).

#### Vital capacity

All students were instructed to assess vital capacity using a spirometer in a quiet environment. The test was repeated twice for each student and the better performance from these two tests was recorded.

#### 50-M run

All students were instructed to run as fast as possible in a straight line on a 50-m track to assess sprint speed. The test was performed only once for each student, and the time of the 50-m sprint was recorded to the nearest 0.1 s.

#### Long-jump

All participants were instructed to push off with both feet behind a take-off line and jump forward as far as possible. The distance between the take-off line and the nearest landing point was measured using cm. The test was repeated twice for each participant and the better performance from these two tests was recorded.

#### Sit-reach

All participants were instructed to reach forward with their hands as far as possible along a measuring line in a seated position while fully extending both knees and placing feet firmly against vertical support to assess flexibility; the distance was measured to the nearest 0.1 cm. The test was repeated twice for each participant and the better performance from these two tests was recorded.

#### 800-M run and 1,000-m run

Girls and boys were instructed to run as fast as they could along a track line for 800 m and 1,000 m, respectively, to assess aerobic fitness. Participants who were unable to perform the test or had to stop for rest during the test were allowed to walk or jog. The test was performed only once for each participant, and the time of the run was recorded to the nearest 0.1 s.

#### 1-min-sit-up

All girls were instructed to perform 1-min-sit-up test to assess abdominal muscle strength. Laying with knee bent, feet flat on a floor mat, hands placed on the back of the head, and fingers interlocked with each other were required during the test. A complete sit-up movement refers to elevating the trunk until the elbow made contact with thighs and then lowering the trunk until shoulders blades touched the mat. The test was performed only once for each girl, and the number of sit-ups during 1 min was recorded.

#### Pull-up

All boys were instructed to perform a pull-ups test to assess upper-body strength. Grasping an overhead bar by an overhand grip and leaving the ground with both feet were required during the test. A complete pull-up movement refers to pulling the body up using the arms until the chin was above the top of the bar and then lowering the body to the starting position with extended arms. Boys were encouraged to repeat this movement as many times as possible and the number of completed movements was recorded.

#### Regional sources of students

The regional sources of students were also obtained from the website of CNSPFD, and the population distribution and administrative division were as follows: northeast (3,338), north (7,229), central (57,934), south (6,454), east (14,184), northwest (6,500) and southwest (7,433), respectively.

### Statistical analysis

**Figure 1:**
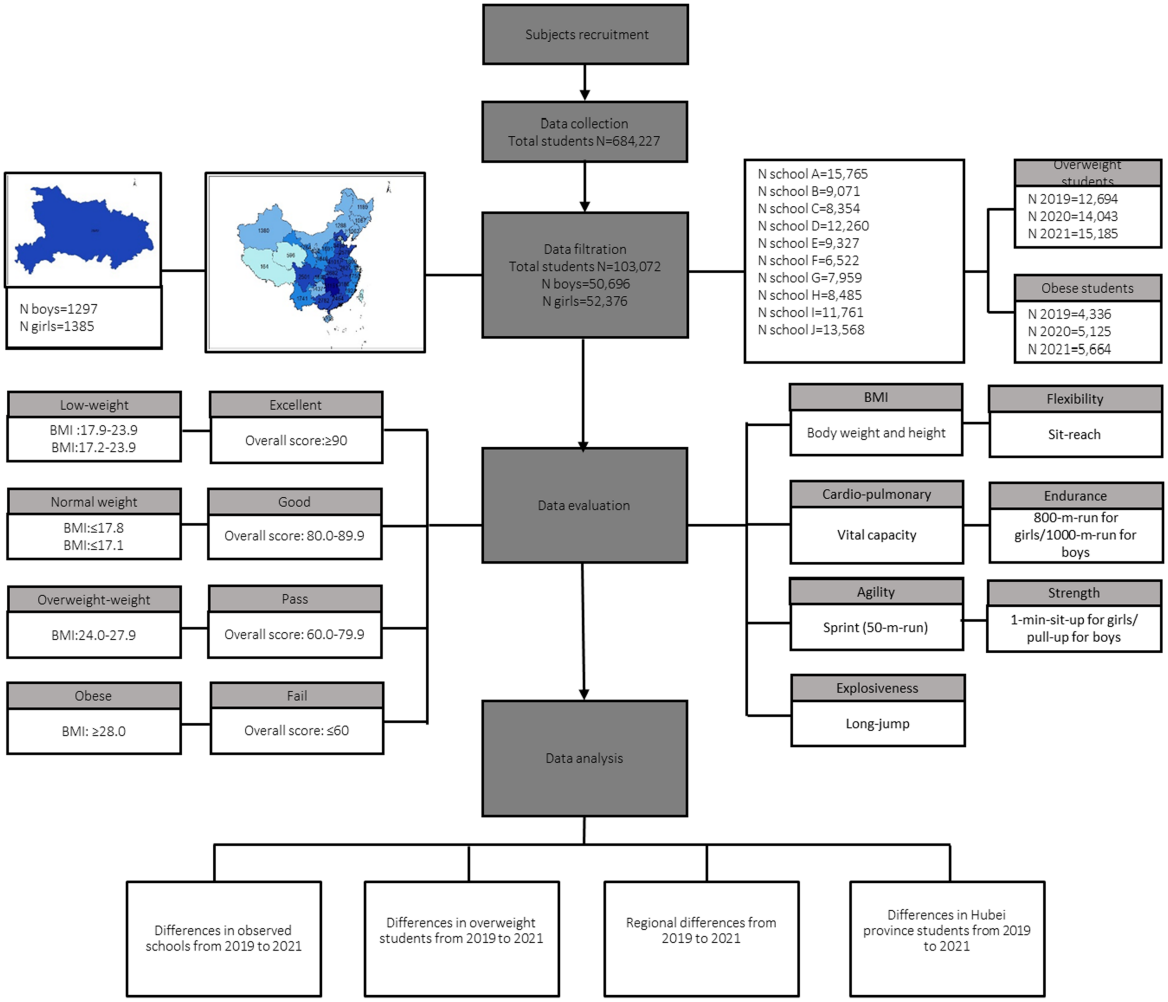
Study flowchart of data collection, filtration, evaluation and analysis.

Means, standard deviations, frequencies, and percentages were calculated to describe physical fitness indicators in both overall subjects and overweight and obesity subjects, grouped by gender and tested year (2019, 2020, and 2021). The Chi-square test was used to measure the differences of physical fitness distribution between overall and overweight students before and after pandemic. RMANOVA and multiple-comparison were performed to examine the differences in changes in physical fitness parameters’ testing-scores before and after pandemic. Two-way ANOVA was applied to horizontally compare the differences in physical fitness indexes among students in the same school year. Statistical significance was set at *p* < 0.05 or *p* < 0.01 and all statistical analyses were conducted using IBM SPSS statistics 17.0. All the analyzed Chi-square value (χ^2^), *F*-value and *T*-value were shown. The regional variations maps were plotted with ArcGIS 10.4, and radar graphs were performed in Excel 2010. The procedures were in accordance with the Declaration of Helsinki. All the data were approved by Hunan Students’ Physical Fitness Test Data Management Center, and formal consent was obtained from participants before the information was stored in CNSPFD. Figure 1 showed the overall study flow of the present study.

## Results

### Descriptive statistics of overall physical fitness in 2019–2021

Table 2 presents the counts and ranked distribution of overall physical fitness assessments of students from 10 DFC universities and colleges in 2019–2021. The overall trend of the ‘excellent/good’ ratio showed an apparent increase (*p* < 0.01) in 2020 and followed by a sharp decline (*p* < 0.01) in 2021 reaching the lowest point at 8.97%, whereas the total “fail” percentage was exhibited a continuous increase year by year peaking at 12.94% in 2021. The “excellent” ratios in School B and E were consistently above average among the three consecutive years. The “fail” ratios of 2021 in School B, C, and I were extremely higher compared to both 2019 and 2020. Accordingly, the trend of the overweight and obesity ratio was significantly increased year by year (*p* < 0.01) reaching the highest values of 14.73 and 5.50% in 2021, respectively. School A and D had above-average overweight and obesity ratio in 2019–2021.

**Table 2:** Frequency number and ranked distribution of overall physical fitness assessments grouped by school and surveyed-year.

School	Year	Excellent/good				Pass		Fail				Overweight				Obesity			
		N	%	χ^2^ vs. 2019	χ^2^ vs. 2020	N	%	N	%	χ^2^ vs. 2019	χ^2^ vs. 2020	N	%	χ^2^ vs. 2019	χ^2^ vs. 2020	N	%	χ^2^ vs. 2019	χ^2^ vs. 2020
A	2019	1,588	10.07	**/**	**/**	12,780	81.07	1,397	8.86	**/**	**/**	2,411	15.29	**/**	**/**	907	5.75	**/**	**/**
(N = 15,765)	2020	2,838	18.00**	**410.68**	**/**	11,069	70.21	1,858	11.79**	**72.81**	**/**	2,539	16.11*	**3.93**	**/**	1,070	6.79**	**14.34**	**/**
	2021	2,253	14.29**##	**131.1**	**80.17**	12,048	76.42	1,464	9.29##	1.73	**52.23**	2,473	15.69	0.93	1.03	1,018	6.46**	**6.82**	1.39
B	2019	1,345	14.83	**/**	**/**	7,295	80.42	431	4.75	**/**	**/**	1,043	11.5	**/**	**/**	305	3.36	**/**	**/**
(N = 9,071)	2020	1,955	21.55**	**137.83**	**/**	6,568	72.41	548	6.04**	**14.78**	**/**	1,378	15.19**	**53.49**	**/**	448	4.94**	**28.33**	**/**
	2021	1,192	13.14**##	**10.73**	**223.82**	6,604	72.8	1,275	14.06**##	**460.89**	**322.31**	1,473	16.24**	**85.32**	3.76	518	5.71**#	**57.75**	**5.36**
C	2019	1,061	12.7	**/**	**/**	6,313	75.57	980	11.73	**/**	**/**	714	8.55	**/**	**/**	208	2.49	**/**	**/**
(N = 8,354)	2020	1,331	15.93**	**35.57**	**/**	6,348	75.99	675	8.08**	**62.39**	**/**	556	6.66 **	**21.27**	**/**	147	1.76**	**10.71**	**/**
	2021	371	4.44 **##	**363.64**	**602.9**	6,386	76.44	1,597	19.12**##	**174.67**	**433.04**	1,300	15.56**##	**193.87**	**335.51**	500	5.99**##	**125.76**	**200.35**
D	2019	1,557	12.7	**/**	**/**	9,413	76.78	1,290	10.52	**/**	**/**	1,782	14.54	**/**	**/**	705	5.75	**/**	**/**
(N = 12,260)	2020	1,114	9.09**	**82.46**	**/**	9,313	75.96	1,833	14.95**	**127.3**	**/**	1,919	15.65*	**5.97**	**/**	847	6.91**	**13.87**	**/**
	2021	1,293	10.55**##	**27.67**	**14.76**	9,298	75.84	1,669	13.61**##	**55.21**	**15.08**	2,172	17.72**##	**45.86**	**18.78**	935	7.63**#	**34.57**	**4.69**
E	2019	1,601	17.17	**/**	**/**	7,211	77.31	515	5.52	**/**	**/**	935	10.02	**/**	**/**	288	3.09	**/**	**/**
(N = 9,327)	2020	1,821	19.52**	**17.32**	**/**	6,953	74.55	553	5.93	1.43	**/**	1,053	11.29**	**7.84**	**/**	330	3.54	2.95	**/**
	2021	1,025	10.99**##	**147.04**	**262.72**	7,604	81.53	698	7.48**##	**29.53**	**18.01**	1,200	12.87**##	**37.14**	**10.91**	417	4.47**##	**24.53**	**10.56**
F	2019	847	12.99	**/**	**/**	5,317	81.52	358	5.49	**/**	**/**	755	11.58	**/**	**/**	243	3.73	**/**	**/**
(N = 6,522)	2020	803	12.31	1.34	**/**	5,102	78.23	617	9.46**	**74.36**	**/**	764	11.71	0.06	**/**	289	4.43*	**4.15**	**/**
	2021	514	7.88**##	**90.97**	**70.54**	5,435	83.33	573	8.79**	**53.47**	1.79	841	12.89	5.28	4.21	295	4.52*	**5.24**	0.06
G	2019	880	11.06	**/**	**/**	6,520	81.92	559	7.02	**/**	**/**	879	11.04	**/**	**/**	338	4.25	**/**	**/**
(N = 7,959)	2020	1,075	13.51**	**22.17**	**/**	6,219	78.14	665	8.36**	**9.94**	**/**	1,200	15.08**	**55.47**	**/**	409	5.14**	**7.02**	**/**
	2021	483	6.07**##	**126.46**	**249.35**	6,725	84.5	751	9.44**#	**30.66**	**5.73**	1,048	13.17**##	**16.45**	**11.62**	421	5.29**	**9.44**	0.18
H	2019	1,190	14.02	**/**	**/**	6,681	78.74	614	7.24	**/**	**/**	830	9.78	**/**	**/**	256	3.02	**/**	**/**
(N = 8,485)	2020	1,297	15.29*	**5.39**	**/**	6,461	76.15	727	8.57**	**10.34**	**/**	897	10.57	2.89	**/**	256	3.02	0	**/**
	2021	387	4.56**##	**450.77**	**545.92**	7,230	85.21	868	10.23**##	**47.7**	**13.76**	961	11.33**	**10.71**	2.48	220	2.59	2.8	2.8
I	2019	815	6.93	**/**	**/**	9,160	77.88	1,786	15.19	**/**	**/**	1,573	13.37	**/**	**/**	604	5.14	**/**	**/**
(N = 11,761)	2020	577	4.91**	**43.25**	**/**	8,916	75.81	2,268	19.28**	**69.24**	**/**	1,630	13.86	1.17	**/**	691	5.88*	**6.19**	**/**
	2021	237	2.02**##	**332.44**	**147.11**	8,471	72.03	3,053	25.96**##	**417.66**	**149.67**	1,589	13.51	0.09	0.61	706	6.00**	**8.41**	0.17
J	2019	1,671	12.32	**/**	**/**	10,350	76.28	1,547	11.4	**/**	**/**	1,772	13.06	**/**	**/**	482	3.58	**/**	**/**
(N = 13,568)	2020	1,678	12.37	0.02	**/**	10,253	75.57	1,637	12.07	2.88	**/**	2,107	15.53**	**33.76**	**/**	638	4.74**	**22.66**	**/**
	2021	1,490	10.98**##	**11.73**	**12.63**	10,686	78.76	1,392	10.26**##	**9.17**	**22.31**	2,128	15.68**	**37.95**	0.12	634	4.71**	**21.59**	0.01
Total	2019	12,555	12.18	**/**	**/**	81,040	78.62	9,477	9.19	**/**	**/**	12,694	12.32	**/**	**/**	4,336	4.21	**/**	**/**
(N = 103,072)	2020	14,489	14.06**	**159.19**	**/**	77,202	74.9	11,381	11.04**	**193.37**	**/**	14,043	13.62**	**78.21**	**/**	5,125	4.97**	**68.96**	**/**
	2021	9,245	8.97**##	**562.01**	**1309.41**	80,487	78.09	13,340	12.94**##	**735.42**	**176.39**	15,185	14.73**##	**257.38**	**51.99**	5,664	5.50**##	**185.35**	**28.41**

*p < 0.05 and **p < 0.01, compared to 2019; #*p* < 0.05 and ##*p* < 0.01, compared to 2020; only the ratios of ranked “fail,” “excellent/good,” “overweight,” and “obesity” were evaluated. Bold values refers χ^2^ > 3.84.

### Overweight and obesity prevalence in 2019–2021

As summarized in Table 3, the overweight and obesity ratio in boys showed a consistent upward trend from 2019 to 2021 peaking at 29.25% in 2021, and compared to 2019, there was a newly increased overweight ratio of 4.51% in boys. In contrast the situation for girls initially showed a slight decline, followed by a modest increase with the ratio ranging from 10.70% (in 2019) to 11.48% (in 2021). The percentage of scored ‘excellent/good’ among overweight and obesity students was very low, while the percentage of scored ‘fail’ was oppose. The ‘fail’ ratio in both in overweight and obesity showed a rising trend, with the highest being 65.27% (overweight) and 27.92%(obesity) among boys in 2021, significantly higher than that in 2019 (*p* < 0.01). Similarly, among girls, the ‘fail’ ratio reached 11.30% (overweight) and 34.29%(obesity) in 2021, exceeding the levels of the previous 2 years (*p* < 0.01). Compared to girls, the prevalence of overweight and obesity of boys was remarkably higher (*p* < 0.01).

**Table 3:** Frequency number and ranked distribution of overall physical fitness assessments grouped by gender and surveyed-year among overweight and obesity students.

Overweight/Obesity	Gender	Year	Total			Excellent/good				Pass		Fail			
			N	%	χ^2^ vs. girls	N	%	χ^2^ vs. 2019	χ^2^ vs. 2020	N	%	N	%	χ^2^ vs. 2019	χ^2^ vs. 2020
Overweight	boys	2019	8,216	16.20**	**1398.52**	124	1.51	**/**	**/**	6,123	74.53	1,969	23.97	**/**	**/**
		2020	9,874	19.46**	**2894.79**	230	2.33**	**15.72**	**/**	6,897	69.85	2,747	27.82**	**34.58**	**/**
		2021	10,504	20.71**	**2837.69**	181	1.72##	1.32	**9.46**	7,390	70.35	2,933	27.92**	**37.35**	0.03
	girls	2019	4,478	8.56	/	229	5.11	**/**	**/**	3,876	86.56	373	8.33	**/**	**/**
		2020	4,169	7.96	/	226	5.42	0.41	**/**	3,570	85.63	373	8.95	1.04	**/**
		2021	4,681	8.94	/	162	3.46**##	**15.30**	**20.21**	3,990	85.24	529	11.3**##	**22.76**	**13.35**
Obesity	boys	2019	3,214	6.34**	**1126.35**	5	0.16	**/**	**/**	1,374	42.75	1,835	57.09	**/**	**/**
		2020	4,052	7.99**	**1922.17**	6	0.15	0.01	**/**	1,481	36.55	2,565	63.3**	**28.92**	**/**
		2021	4,334	8.54**	**1787.32**	11	0.25	0.84	1.16	1,494	34.47	2,829	65.27**	**52.31**	3.55
	girls	2019	1,122	2.14	/	13	1.16	**/**	**/**	821	73.17	288	25.67	**/**	**/**
		2020	1,073	2.05	/	7	0.65	1.56	**/**	770	71.76	296	27.59	1.03	**/**
		2021	1,330	2.54	/	16	1.20	0.01	1.90	858	64.51	456	34.29**##	**21.38**	**12.40**
Total	boys	2019	11,430	22.53**	**2624.63**	129	1.13	**/**	**/**	7,497	65.59	3,804	33.28	**/**	**/**
		2020	13,926	27.45**	**5174.18**	236	1.69**	**14.18**	**/**	8,378	60.16	5,312	38.14**	**64.49**	**/**
		2021	14,838	29.25**	**5038.96**	192	1.29##	1.46	**7.87**	8,884	59.87	5,762	38.83**	**85.95**	1.44
	girls	2019	5,600	10.70	/	242	4.32	**/**	**/**	4,697	83.88	661	11.80	**/**	**/**
		2020	5,242	10.01	/	233	4.44	0.10	**/**	4,340	82.79	669	12.76	2.31	**/**
		2021	6,011	11.48	/	178	2.96**##	**15.38**	**17.52**	4,848	80.65	985	16.39**##	**50.05**	**29.34**

*p < 0.05 and **p < 0.01, compared to 2019 and compared to girls; #*p* < 0.05 and ##*p* < 0.01, compared to 2020; only the ratios of ranked “excellent” and “fail,” were evaluated. Bold values refers χ^2^ > 3.84.

### Distribution of physical fitness indicator score in overweight and obesity students

**Figure 2:**
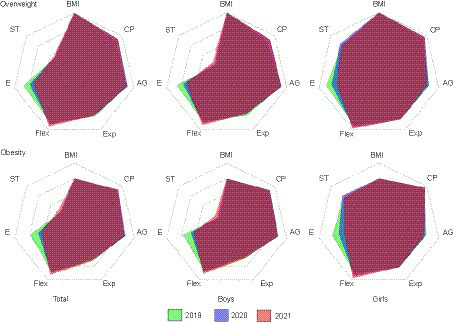
The distribution and change of physical fitness indicator score in overweight and obesity students from 2019 to 2021 (CP, Cardio-pulmonary; AG, Agility; Exp, Explosiveness; Flex, Flexibility; E, Endurance; ST, Strength).

Figure 2 showed the individual physical fitness index scoring among overweight and obesity students. As depicted in the radar chart, the development of college students’ physical fitness was unbalanced, and the score of strength in boys and endurance quality in all was the lowest among all the physical fitness indicators, and the endurance performance worsened from 2019 to 2021 both among the overweight and obesity students.

### Differences of scoring among overweight and obesity students who failed the test in 2021 relative to that in 2019

Table 4 presented the differences in standardized scoring among overweight and obesity students who failed the test in 2021 compared to that in 2019. Except for the vital capacity and sit-reach for both boys and girls, the scores of BMI, 50-m run, long-jump, 800-m-run/1000-m-run, and 1 min-sit-up/pull-up not only showed a negatively increase in 2021 but also were significantly different from those in 2020 (*p* < 0.05).

**Table 4:** The differences of standardized scoring among overweight and obesity students who failed the test in 2021 relative to that in 2019.

Overweight/Obesity	Gender	Year	BMI (kg/m^2^)	Vital capacity(ml)	50-m run(s)	Long-jump(m)	Sit-reach(cm)	800-m-run/	1-min-sit-up/	Overall
								1,000-m-run(s)	Pull-up(n)	
Overweight	boys (N = 2,923)	2020	−0.04 ± 0.00	0.03 ± 0.00	−0.01 ± 0.00	−0.03 ± 0.00	0.02 ± 0.00	−0.12 ± 0.00	−0.01 ± 0.00	−0.03 ± 0.00
		2021	−0.09 ± 0.00**	0.01 ± 0.00**	−0.05 ± 0.00**	−0.10 ± 0.00**	0.04 ± 0.00	−0.27 ± 0.00**	−0.05 ± 0.00**	−0.09 ± 0.00**
		*T*	15.70	3.62	9.17	10.45	−1.92	27.17	6.64	24.93
	girls (N = 555)	2020	−0.01 ± 0.00	0.02 ± 0.01	−0.02 ± 0.01	0.00 ± 0.01	0.04 ± 0.01	−0.15 ± 0.01	0.02 ± 0.01	−0.03 ± 0.00
		2021	−0.09 ± 0.01**	−0.01 ± 0.01*	−0.13 ± 0.01**	−0.08 ± 0.01**	0.03 ± 0.01	−0.39 ± 0.01**	−0.07 ± 0.01**	−0.13 ± 0.00**
		*T*	10.80	2.28	8.31	5.64	0.14	17.68	5.72	18.84
Obesity	boys (N = 2,801)	2020	−0.06 ± 0.00	0.03 ± 0.00	−0.01 ± 0.00	−0.03 ± 0.00	0.01 ± 0.00	−0.12 ± 0.00	0.01 ± 0.00	−0.03 ± 0.00
		2021	−0.14 ± 0.00**	0.02 ± 0.00*	−0.04 ± 0.00**	−0.08 ± 0.01**	0.03 ± 0.00**	−0.24 ± 0.00**	−0.01 ± 0.00**	−0.08 ± 0.00**
		*T*	21.24	2.06	7.74	6.06	−3.22	19.16	3.04	19.31
	girls (N = 483)	2020	−0.02 ± 0.01	0.03 ± 0.01	−0.02 ± 0.01	0.01 ± 0.01	0.03 ± 0.01	−0.13 ± 0.01	0.00 ± 0.01	−0.02 ± 0.00
		2021	−0.15 ± 0.01**	0.02 ± 0.01	−0.11 ± 0.01**	−0.06 ± 0.01**	0.04 ± 0.01	−0.37 ± 0.01**	−0.07 ± 0.01**	−0.12 ± 0.00**
		*T*	13.59	0.60	6.01	4.47	−1.52	15.02	4.49	14.83
Total	boys (N = 5,724)	2020	−0.05 ± 0.00	0.03 ± 0.00	−0.01 ± 0.00	−0.03 ± 0.00	0.02 ± 0.00	−0.12 ± 0.00	0.00 ± 0.00	−0.03 ± 0.00
		2021	−0.11 ± 0.00**	0.01 ± 0.00**	−0.04 ± 0.00**	−0.09 ± 0.00**	0.03 ± 0.00**	−0.26 ± 0.00**	−0.03 ± 0.00**	−0.08 ± 0.00**
		*T*	26.07	4.04	11.89	11.58	−3.59	32.34	6.88	31.08
	girls (N = 1,038)	2020	−0.01 ± 0.00	0.03 ± 0.01	−0.02 ± 0.01	0.00 ± 0.01	0.03 ± 0.01	−0.14 ± 0.01	0.01 ± 0.01	−0.03 ± 0.00
		2021	−0.11 ± 0.00**	0.01 ± 0.01*	−0.12 ± 0.01**	−0.07 ± 0.01**	0.04 ± 0.01	−0.38 ± 0.01**	−0.07 ± 0.01**	−0.13 ± 0.00**
		*T*	17.11	2.07	10.18	7.15	−0.94	23.09	7.26	23.71

*p < 0.05 and **p < 0.01.

### Regional differences of physical health among college students in 2019–2021

#### Regional differences in ‘excellent/ good’, ‘fail’, ‘overweight’ and ‘obesity’ rate of physical fitness

**Figure 3:**
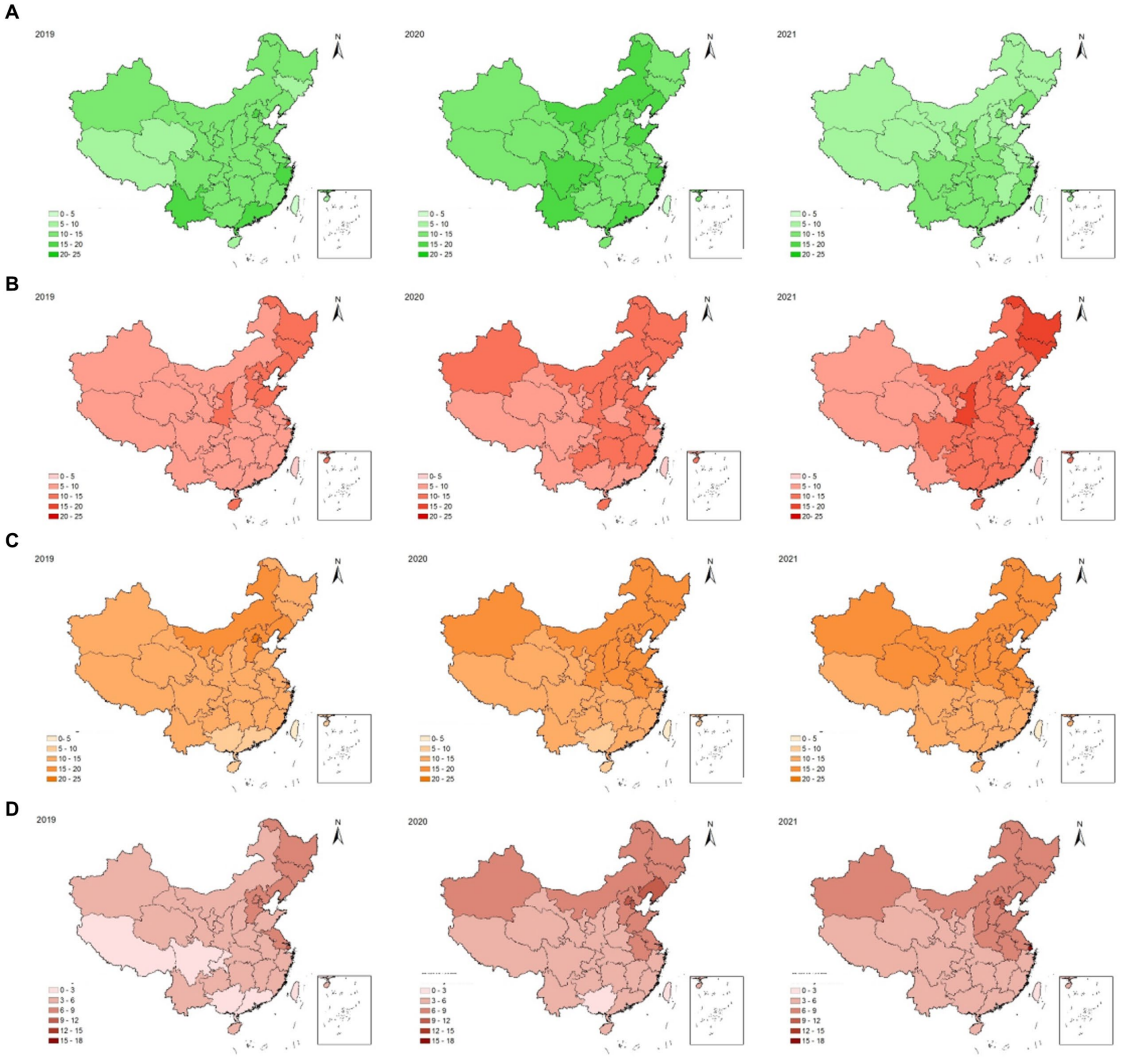
The regional distribution and change of ‘excellent and good’, ‘fail’, ‘overweight’ and ‘obesity’ rate of physical fitness from 2019 to 2021(**A**: ‘excellent/ good’ rate; **B**: ‘fail’ rate; **C**: ‘overweight’ rate; **D**: ‘obesity’ rate).

With regard to the ‘excellent /good’, ‘fail’, ‘overweight’ and ‘obesity’ rate (data shown in Figure 3), over the study period, the proportion of scored ‘excellent /good’ showed an upward trend from 2019 to 2020, followed by a downward trend from 2020 to 2021. Most areas showed a relatively high ‘excellent/ good’ rate in 2019.In 2020, 1 year after COVID-19 outbreak, the ‘excellent/ good’ ratio had a slight increase in all regions, while up to 2021, the number of provinces with a high proportion decreased. Students performed ‘excellent/good’ in both 2019 and 2020 mainly from Beijing, Guangdong and Zhejiang which are developed regions in China. The fail rate presented a basic trend of increasing year by year, and it shifted from northern and northeastern provinces to southern areas from 2019 to 2021. Students from northeastern provinces such as Heilongjiang, Jilin and Liaoning exhibited a higher proportion, while those from western regions showed a relatively lower proportion. The overweight and obesity rate showed an increasing trend from 2019 to 2021. The high proportion of overweight and obesity was mainly appeared in northern, northeastern and eastern provinces (Hebei, Beijing, Tianjin, Inner Mongolia, Heilongjiang, Jilin, Liaoning, Jiangsu and Shanghai) in 2019 and in the following 2 years, it expanded nationwide and moved southward.

### Regional distribution of overweight and obesity students in 2018th and 2019th who failed the standard test

**Figure 4:**
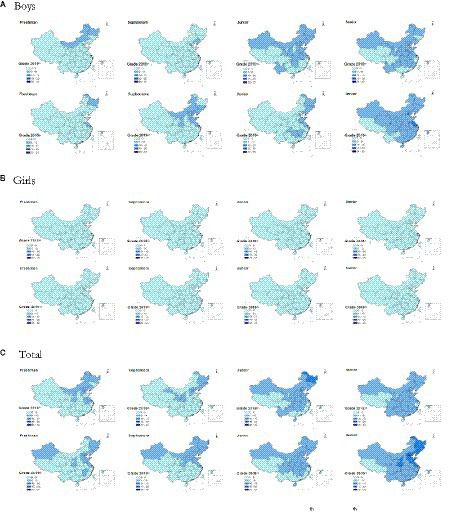
The regional distribution of overweight and obesity students of grade 2018th and 2019th who failed the *standard* test among four academic years (**A**: Boys; **B**: Girls; **C**: Total).

As shown in Figure 4, the regional distribution of overweight and obesity in freshmen who also failed the test mainly included Inner Mongolia, Heilongjiang, Liaoning, Hebei, Beijing, Shanxi, Jiangsu, and Anhui in 2018, and Xinjiang, Shanxi, Heilongjiang, Jilin, Liaoning, Hebei, Beijing, Shandong, Jiangsu, and Jiangxi in 2019. In 2019 when sophomore students of grade 2018th, the ratio and distribution area of this group decreased, while the situation was the opposite in junior (in 2020) and senior (in 2021). In 2022, when senior students of grade 2019th, the regions with a high ratio and distribution were spread nationwide except for Tibet and the most affected areas including Heilongjiang, Jilin, and Liaoning in northeast, Beijing, Hebei, Shandong, Jiangsu, and Shanghai in east, as well as Shanxi in central north. No matter whether it was the class of 2021st or 2022nd, the Ningxia Hui Autonomous Region processed the lowest proportion of college students with physical disadvantage in north China, and students in Yunnan showed a steadily lower proportion of overweight and obesity. The distribution of girls in this group was quite even, while the performance in boys was more severe.

### Differences of scoring among overweight and obesity students in grade 2018th and 2019th who failed the test when graduate relative to those when freshman

As shown in Table 5, in grade 2018th, all the physical parameters showed a positively increase in 2019, while some of these increases significantly decreased in both 2020 and 2021, including BMI, 50-m run, 800-m-run/1000-m-run, and the overall scorings (*p* < 0.01). Different from grade 2018th, the BMI, 50-m run, long-jump, 800-m-run/1000-m-run, and overall scorings already showed a decrease in grade 2019th, and Upon in 2022, all the physical parameters were detected to have a negative increase compared to those in 2021 (*p* < 0.01).

**Table 5:** The differences of standardized scoring among two generations of overweight and obesity graduates who failed the test relative to that when freshmen.

Grade	Year	BMI (kg/m^2^)	Vital capacity(ml)	50-m run(s)	Long-jump(m)	Sit-reach(cm)	800-m-run/	1-min-sit-up/	Overall
2018th	2019	0.02 ± 0.00	0.09 ± 0.01	0.07 ± 0.01	0.13 ± 0.01	0.03 ± 0.01	0.48 ± 0.01	0.10 ± 0.005	0.06 ± 0.00
	2020	−0.04 ± 0.00**	0.05 ± 0.00**	−0.02 ± 0.00**	000 ± 0.01**	0.02 ± 0.01	−0.11 ± 0.01**	0.00 ± 0.006**	−0.02 ± 0.00**
	2021	−0.04 ± 0.00**	0.07 ± 0.01##	−0.02 ± 0.00**	0.01 ± 0.01**#	0.06 ± 0.01**##	−0.13 ± 0.01**##	0.02 ± 0.01**##	−0.02 ± 0.00**
	*P*_2019vs2020_	0.00	0.00	0.00	0.00	1.00	0.00	0.00	0.00
	*P*_2019vs2021_	0.00	0.26	0.00	0.00	0.00	0.00	0.00	0.00
	*P*_2020vs2021_	0.21	0.00	1.00	0.01	0.00	0.00	0.00	1.00
2019th	2020	−0.03 ± 0.00&&	0.05 ± 0.00&&	−0.08 ± 0.00&&	−0.01 ± 0.00&&	0.03 ± 0.00&&	−0.08 ± 0.00&&	0.03 ± 0.00&&	−0.01 ± 0.00
	2021	−0.03 ± 0.00&&	0.04 ± 0.00**&&	−0.15 ± 0.00**&&	−0.01 ± 0.00&&	0.05 ± 0.00**&&	−0.15 ± 0.00**	0.05 ± 0.00**&&	−0.02 ± 0.01**&&
	2022	−0.09 ± 0.00**##&&	−0.02 ± 0.00**##	−0.19 ± 0.00**##&&	−0.06 ± 0.00**##&&	−0.03 ± 0.00**##&	−0.19 ± 0.00**##&&	−0.14 ± 0.00**##&&	−0.10 ± 0.00**##&&
	*P*_2019vs2020_	1.00	0.00	0.00	0.90	0.00	0.00	0.00	0.00
	*P*_2019vs2021_	0.00	0.00	0.00	0.00	0.00	0.00	0.00	0.00
	*P*_2020vs2021_	0.00	0.00	0.00	0.00	0.00	0.00	0.00	0.00
	*T*_2020(2019_^th^_) vs.2019(2018_^th^_)_	7.09	−7.36	−5.79	7.92	−14.73	105.25	15.73	−0.61
	*T*_2021(2019_^th^_) vs. 2020(2018_^th^_)_	−8.08	−3.26	−3.97	−4.95	−8.12	0.74	−13.12	−10.51
	*T*_2022(2019_^th^_) vs. 2021(2018_^th^_)_	−6.63	−1.90	5.93	−3.40	2.54	7.96	6.09	5.51

*p < 0.05 and **p < 0.01, compared to 2019 in 2018th and compared to 2020 in 2019th; #*p* < 0.05 and ##*p* < 0.01, compared to 2020 in 2018th and compared to 2021 in 2019th; &*p* < 0.05 and &&*p* < 0.01, compared to grade 2018th.

### Disparity of physical fitness among students from Hubei Province in 2019–2021

#### Descriptive statistics of overall physical fitness in 2019–2021

As shown in Table 6, the ratio of total ‘excellent/good’ in Hubei province students was 10.85% in 2019, 14.43% in 2020, and 10.07% in 2021, and the total ‘fail’ ratio achieved 8.99, 10.33 and 12.90%.for the corresponding years. The proportion of overall ‘excellent/good’ followed a trend of initially increasing firstly after and then declining (*p* < 0.01), while the total ‘fail’ ratio steadily improved year by year (*p* < 0.01). There were no significant differences in the proportion of overweight and obesity among the total population and girls from 2019 to 2021except for boys’ overweight proportion which slightly increased in 2021 compared to those in 2019.

**Table 6:** Number and ranked distribution of overall fitness in Hubei province students by gender and test year.

Gender	Year	Excellent/Good				Pass		Fail				Overweight				Obesity			
		N	%	χ^2^ vs. 2019	χ^2^vs. 2020	N	%	N	%	χ^2^vs. 2019	χ^2^ vs. 2020	N	%	χ^2^ vs. 2019	χ^2^ vs. 2020	N	%	χ^2^vs. 2019	χ^2^vs. 2020
Boys (N = 1,297)	2019	66	5.09	**/**	**/**	1,053	81.19	178	13.72	**/**	**/**	221	17.04	**/**	**/**	89	6.86	**/**	**/**
	2020	90	6.94*	**3.93**	**/**	993	76.56	214	16.5*	**3.89**	**/**	256	19.74	3.15	**/**	105	8.1	1.43	**/**
	2021	79	6.09	1.23	0.77	961	74.09	257	19.81**#	**17.24**	**4.80**	269	20.74*	**5.80**	0.4	105	8.1	1.43	0.00
Girls (N = 1,385)	2019	225	17.35	**/**	**/**	1,097	79.21	63	4.55	**/**	**/**	140	10.11	**/**	**/**	35	2.53	**/**	**/**
	2020	297	22.9**	**12.24**	**/**	1,025	74.01	63	4.55	0.00	**/**	111	8.01	3.68	**/**	41	2.96	0.49	**/**
	2021	191	14.73##	3.27	**27.95**	1,105	79.78	89	6.43*#	**4.71**	**4.71**	115	8.3	2.70	0.08	35	2.53	0.00	0.49
Total (N = 2,682)	2019	291	10.85	**/**	**/**	2,150	80.16	241	8.99	**/**	**/**	361	13.46	**/**	**/**	124	4.62	**/**	**/**
	2020	387	14.43**	**15.56**	**/**	2,018	75.24	277	10.33	2.77	**/**	367	13.68	0.06	**/**	146	5.44	1.89	**/**
	2021	270	10.07##	0.88	**23.74**	2,066	77.03	346	12.90**#	**21.09**	**8.65**	384	14.32	0.82	0.45	140	5.22	1.02	0.13

*p < 0.05 and **p < 0.01, compared to 2019; #*p* < 0.05 and ##*p* < 0.01, compared to 2020; only the ratios of ranked “fail,” “excellent/good,” “overweight,” and “obesity” were evaluated. Bold values refers χ^2^ > 3.84.

#### Differences of standardized scoring among overweight and obesity students in 2018th and 2019th who failed the test in 2021

As shown in Table 7, the overall scoring growths in both class of 2021st and 2022nd were measured to be a negatively increase (*p* < 0.01). For grade 2019th, except for the vital capacity and sit-reach, the remaining parameters were obviously decreased when compared to those in both 2020 and 2021 (*p* < 0.01).

**Table 7:** The differences of standardized scoring among two generations of overweight and obesity graduates who failed the test relative to that when freshmen in Hubei province.

Grade	Year	BMI (kg/m^2^)	Vital capacity(ml)	50-m run(s)	Long-jump(m)	Sit-reach(cm)	800-m-run/	1-min-sit-up/	Overall
2018th	2019	0.01 ± 0.02	0.22 ± 0.06&	0.14 ± 0.05	0.20 ± 0.055	−0.01 ± 0.04	0.50 ± 0.05	0.16 ± 0.04	0.10 ± 0.02
	2020	−0.06 ± 0.02	−0.04 ± 0.03**	−0.04 ± 0.03*	−0.04 ± 0.042*	−0.03 ± 0.03	−0.13 ± 0.05**	−0.06 ± 0.04**	−0.06 ± 0.02**
	2021	−0.10 ± 0.02**	0.07 ± 0.03#	−0.04 ± 0.03*	−0.01 ± 0.046	0.02 ± 0.04	−0.08 ± 0.05**	0.05 ± 0.04	−0.02 ± 0.02**#
	*P*_2019vs2020_	0.08	0.00	0.04	0.02	1.00	0.00	0.01	0.00
	*P*_2019vs2021_	0.00	0.10	0.02	0.07	1.00	0.00	0.23	0.00
	*P*_2020vs2021_	0.10	0.03	1.00	1.00	0.25	0.85	0.06	0.03
2019th	2020	−0.04 ± 0.01	0.07 ± 0.02	0.00 ± 0.01	−0.02 ± 0.03	0.04 ± 0.02	−0.11 ± 0.02&&	0.06 ± 0.03	−0.01 ± 0.01
	2021	−0.04 ± 0.01	0.05 ± 0.02	0.00 ± 0.01	0.01 ± 0.03	0.04 ± 0.02	−0.12 ± 0.02	0.08 ± 0.03&	0.00 ± 0.01&&
	2022	−0.04 ± 0.02**##	0.02 ± 0.02	−0.18 ± 0.03**##&	−0.12 ± 0.03**##	0.02 ± 0.02	−0.37 ± 0.03**##	−0.10 ± 0.03**##	−0.14 ± 0.01**##
	*P*_2019vs2020_	0.10	0.83	1.00	0.58	1.00	1.00	1.00	1.00
	*P*_2019vs2021_	0.00	0.06	0.00	0.00	0.63	0.00	0.00	0.00
	*P*_2020vs2021_	0.00	0.35	0.00	0.00	0.63	0.00	0.00	0.00
	*T*_2020(2019_^th^_) vs.2019(2018_^th^_)_	1.40	−2.07	0.09	1.56	−1.74	18.49	1.64	0.26
	*T*_2021(2019_^th^_) vs. 2020(2018_^th^_)_	−1.13	−1.54	−1.53	−1.80	−1.19	−1.60	−2.40	−3.47
	*T*_2022(2019_^th^_) vs. 2021(2018_^th^_)_	−1.01	−1.61	2.95	−0.13	−1.32	2.56	0.93	1.67

*p < 0.05 and **p < 0.01, compared to 2019 in 2018th and compared to 2020 in 2019th; #*p* < 0.05 and ##*p* < 0.01, compared to 2020 in 2018th and compared to 2021 in 2019th; &*p* < 0.05 and &&*p* < 0.01, compared to grade 2019th.

## Discussion

*Students’ physical fitness and health declined and the prevalence of overweight and obesity increased*. Regular physical fitness surveillance is widely executed around the world, and Department of Physical Health and Arts Education, Ministry of Education of China, is responsible to collect and monitor the national physical health. Within this process, the health issues of children and adolescents at school age is high-profile. The latest report of the Eighth National Survey on Student Physical Fitness and Health in 2019 (30) showed a general improvement in student physical fitness and health in China while since then, there have been few studies on the physical fitness and health of students, especially during the COVID-19 pandemic and among students from DFC universities and colleges. The present study revealed that 12.94% of students from DFC universities and colleges failed in the physical fitness *standard* test in 2021, which is recorded as the historical highest rate, and only 8.97% performed and ranked in ‘*excellence/good’*. Nerveless, there were persistent and increasing populations of overweight and obesity observed over the course of the 3-year survey, with the highest ratios of these two groups were 14.73 and 5.50% in 2021. Researchers reported that the nutrient intake and physical activity levels of university students decreased with a significant increase in sedentary activity during COVID-19, and the insufficient physical activity was unable to offset the increased sedentary behavior, contributing to the worsening of student physical fitness and health (31–36). The DFC plan, founded in 2015, aims to comprehensively develop the target universities and faculties into world-class and globally-ranked universities by 2050, and the physical fitness and health of students from DFC universities and colleges needs to align with the increased investment and resources allocated to these schools’ development. If there was no global COVID-19 lockdown, the positive trend toward improved student physical fitness and health would continue in China. However, the worse physical fitness and health in students from DFC universities and colleges, to some extent, represented the elite portion of college students nationwide. It is crucial to deeply analyze the parameters of tested *standard* data, comprehensibly explore the reasons behind the decline of students’ physical fitness and health, closely focus on the groups of overweight and obesity students, integrally observe the regional distribution features of the entire student sample and the overweight and obesity students, and especially study the data from Hubei province, the origin of the epidemic.

*The prevalence of overweight and obesity in boys was even higher than that in girls, and the decline in endurance physical fitness was the most prominent*. The cross-sectional study showed that both 2020 and 2021 witnessed a higher prevalence of overweight and obesity in students from DFC universities and colleges compared to 2019. The global prevalence of obesity had increased substantially over the past 40 years, and according to the obesity transition model, a large increase in the prevalence among adults and a small increase among children would be predicted in China (37). Studies proved that the need for social isolation had the effect of causing or worsening obesity and its comorbidities in children and adolescents (38). A clinical study confirmed that obesity increased the risk of hospitalization, intensive care admission, mechanic ventilation requirement, and death among children and adolescents with COVID-19 (39) and another study proved that obesity is associated with an increased risk of COVID-19 mortality due to the interaction of adipocytes with angiotensin-converting enzyme 2 (ACE2) and interleukin 6 (IL-6) (40); the outbreak had exacerbated the overweight and obesity prevalence among students of all school-ages due to social isolation and e-learning (41). Our data firstly reported that the overweight and obesity prevalence in boys (29.25% in 2021) was even worse than that in girls (11.48% in 2021) among students from DFC universities and colleges. Furthermore, compared to 2019, the newly increased 4.51% of overweight boys clearly reflected the influence of the epidemic, to which should be paid attention by the education department when reforming the current physical education and promoting students’ physical fitness and health. Our results are in agreement with previous studies performed in Chinese children and adolescents (aged 6–22 years).The authors showed that the performance of muscular strength (sit-ups and pull ups), flexibility (sit and reach) and vital capacity increased while middle-distance race decreased during the pandemic (42, 43).However, contrary results have been found in adolescent population across many countries (44, 45), where greater reductions were observed in most physical fitness. These contradictory results could be partly influenced by the differences in evaluating physical ability across different countries, and inconsistent participant age stages in previous studies were another determinant. Nonetheless, the principles of the underlying physiological mechanism deserve to be further explored in the future. To delve deeper into the Radar Chart on individual physical fitness index scoring, both overweight and obese students performed worse and worse on the endurance, which was considered as the most important affected aspect due to the epidemic. A study from mild COVID-19 infected athletes confirmed decreased cardiopulmonary exercise test performance among endurance athletes with varied fitness level with poorer VO_2_max and heart rate (46). Besides, the intervention of physical education after returning to school may explain the positive trend of flexibility and strength as well as the maintenance of agility and explosiveness, while the elevated vital capacity could be attributed to the continuous increase in student body weight. Additionally, some of the improvements in physical fitness among these students may be due to the increased awareness of exercise and health during pandemic. Moreover, the decline in the middle-distance run after the pandemic outbreak may be attributed to the increase in BMI, as there is a negative correlation between these two variables, and increasing BMI has been proven to result in a decrease in the performance of endurance running by many studies (47, 48). And lastly, the development of college students’ physical fitness was unbalance, with boys showing weakness in strength and girls exhibiting weakness in endurance. Therefore, school polices and PE teachers should pay more attention to improving students’ weak physical qualities. The epidemic presented both an opportunity and a challenge for health, because on one hand, the public had never been so concerned about their physical activity and its effect on health, and on the other hand, the authorities and education department had taken specialized action to offset the decline of physical activity in students (49).

*All aspects of physical fitness of overweight and obese who failed standard test deteriorated after the pandemic lockdown.* Although not all overweight and obese students were in poor health, in 2021, the prevalence exceeded the levels of the previous 2 years, with 27.92% overweight and 65.27% obesity boys failing the *standard* test, so did the 11.30 and 34.29% in girls. To further study the concrete transformation of physical fitness parameters, we focused on the overweight and obesity students who also failed the test in 2021 and filtered their performance data in 2020 and 2019, and all the values were applied by standardized scoring and represented by the relative differences from 2019. It should be noticed that the DFC universities and colleges mainly cover science and engineering or comprehensive universities, leading to a natural imbalance in the ratio of male and female. However, to our shock, boys were 5-time more likely to be overweight and obese and fail the *standard* test than girls. On one hand, the *standard* measurements did not prevail for boys; on the other hand, the overweight and obesity epidemic of male students from colleges and universities, especially from science and engineering colleges, had also attracted more and more attention academically. Clinically physical examination results showed the prevalence of obesity in boys exceeded that in girls of multiple universities (50–53). Judging from the physical performance, as expected, the scoring of body composition, agility, explosive, endurance, and strength both in 2020 and 2021 was detected a negative increase in boys as well as girls. Meanwhile the scores of cardio-pulmonary function and flexibility were basically maintained, with a slight increase in cardio-pulmonary scoring in 2020 and a negative growth in 2021. Consistent with the overall sample and even more prominent, the decline in endurance was most pronounced in this group, and more severe in females. Hence, we have to think what kind of school physical promoting strategy is suitable to solve the obesity prevalence issue of college students, especially boys, and facilitate their physical health. Based on the above problems, there is a need for more powerful physical education reforms and researches to further study. If schools do not take action immediately, the DFC construction and the elite talent export may be curbed.

*There was a regional polarization between the ‘excellent/good’ rate and the ‘fail rate, and national coverage of overweight and obesity continued to increase and moved southward.* It is conceivable that such variation may reflect geographic disparities in regional economic status, diet structure, lifestyle and the environment of geography and climate. in addition, COVID-19 pandemic was more severe in northern areas, where students’ excise space, sport facilities and outdoor physical activities were restricted due to lockdown, which was another important reason. In a certain sense, health is not only a medical problem, but also an important social problem. The historical experience of human development persuaded the influence of social factors, including but not limited to economic development and the improvement of social environment, on health was far greater than that of medical technology (54, 55). China has a vast territory, and it is divided into seven geographical areas based on its characteristics such as geography and climate conditions, custom and economy status. From north to south and from east to west, these geographical areas include Northeast, North China, Central China, South China, East China, Northwest and Southwest. In this study, according to students’ birth place, we aim to explore the regional differences in their physical fitness status, especially the changes before and after the COVID-19 pandemic outbreak. Coupled with the occurrence of the epidemic, the regional distribution of student physical fitness had been depicted with new features, and due to the local lockdown, the regional epidemic prevention and countermeasures cannot be ignored when studying the students’ physical conditions. Beijing, Guangdong, and Zhejiang were relatively developed regions in China, and the students performed ‘*excellent/good*’ in both 2019 and 2020 were mainly from these areas. The slightly increased ratio of students who performed ‘*excellent/good*’ nationwide once again proved the reverse incentive effect of epidemic on the physical advantaged group, while the overall decline in student physical fitness until 2021 was inevitable reflected by the southward increase in ‘fail’ ratio. So far, the spatial distribution features of college students’ physical fitness were not studied well, it was achievable to apply geography in physical education providing practical experience (56). Consistent with the previous finding in 2020, without the influence of the outbreak, college students’ physical fitness in South China was presented superior to the north, while the western plateau and the northeast exhibited the least developed physical fitness (57, 58). When formulating physical fitness test standards and promoting physical health for college students, especially students from the western and northeast regions of China, the matching and key interventions should be considered by the authorities.

*The regional distribution of overweight and obesity students from class of 2021st and 2022nd who also failed the standard test showed different characteristics with a higher clustering and more areas affected by pandemic observed in the later generation.* Overweight and obesity students who also failed in the physical test were the focus groups in the process of health promotion in colleges, and the regional distribution characteristics of these students’ each academic year were studied. Physical education (PE) curriculum was established and made available to help maintain and improve the student physical condition in colleges; however, as the end of PE class, with the increase in study burden and the enlargement of employment pressure, more and more students might be surrounded by anxiety in the later academic years, especially during graduate year (59). Due to campus PE courses being basically carried out in their first and second year after admission in most of our surveyed schools, it is important, from the health promotion perspective, to encourage the breaking of sedentary time and engagement in sporting activities through school policy and practice recommendations in junior and senior students, besides impacting the natural processes of growth and maturation. Concretely, the physical test was carried out mostly in autumn semester, and the regional distribution of overweight and obesity in freshmen who also failed the test reflected these students’ sources, which mainly included Inner Mongolia, Heilongjiang, Liaoning, Hebei, Beijing, Shanxi, Jiangsu, and Anhui in 2018, and Xinjiang, Shanxi, Heilongjiang, Jilin, Liaoning, Hebei, Beijing, Shandong, Jiangsu, and Jiangxi in 2019. After 1 year of physical intervention on campus, the ratio and distribution area of overweight and obesity students who failed the *standard* test was controlled effectively in 2019 when they were sophomore, while this effect was offset in 2020 for the later generation, mainly due to the outbreak. It is worth noting that during pandemic, the Ningxia Hui Autonomous Region had the fewest physical disadvantage college students in North China, depending on the regional economic policy. Regardless of the impact of the pandemic, due to societal development and excessive energy intake, the physical health of students was declining globally year by year (26, 60–64). The physical health status of the younger generation of DFC students was deteriorating from the class comparison between 2018 and 2019, which implied the urgent need to prioritize physical health. The far-reaching influence of the COVID-19 on student physical health was shown in 2022 among grade 2019th with severest national distribution of overweight and obesity students who also failed the *standard* test, and the most affected areas, including Heilongjiang, Jilin, and Liaoning in northeast, Beijing, Hebei, Shandong, Jiangsu, and Shanghai in east, as well as Shanxi in central north, possessed the higher ratios. No matter before or during pandemic, students in Yunnan steadily showed less portion of overweight and obesity, which might be attributed to the regional characteristics of diet culture and geographical features. The distribution of girls in this group was quite even, while the performance of boys was worried urgently. Thus, when designing specific interventions to promote physical activity and health among college students, the government and related functional departments should pay more attention to the birthplace and grade of students. As a follow-up, we backtracked the relative scoring growth of physical fitness to their performance when they were freshmen in the overweight and obesity students who failed the *standard* in two graduates, and found all the aspects of physical fitness in young generation should be targeted for future interventions, including body composition, cardio-pulmonary function, agility, explosiveness, flexibility, endurance, and strength individually or in combination. The influence of geographical factors needs to be considered comprehensively for the health promotion of these students, and the local governments should also invest more in health after the epidemic. In terms of family and personal factors, only adopting a proper life style and engaging in regular physical exercise could have a positive effect on the physical health of college students (65–67). The exploration and research on integrating physical promotion with family, school, and society needs to be further studied. In China, the spread of the epidemic was associated with population movement and regional joint prevention, and epidemic prevention and control differed between urban and rural areas. Unfortunately, the distribution of urban and rural students was not available in the present study, and the difference in the student physical health status between urban and rural areas before and during the epidemic remained unknown. Moreover, whether a student is infected with COVID-19 is a matter of privacy, the comparison of students’ physical fitness before and after COVID-19 infection was not reported here. Similar to the above, here, we firstly reported the national coverage of overweight and obesity rates of DFC universities and colleges since the outbreak, providing realistic dilemmas for school health promotion.

*The ratio of students who failed in the standard test increased significantly due to the influence of the COVID-19 outbreak* in Hubei*, while the ratio of “excellent/good” increased in 2020 and decreased in 2021.* To look back, the epidemic was undoubtedly a challenge for human life, but also brought with the development opportunity. Our study indicated that students from *Hubei Province*, where the COVID-19 outbreak occurred firstly and severely in China, showed a higher “*fail*” proportion and better performance in most of the individual physical fitness indexes during pandemic years, and the results were in accordance with the national data. It is comforting to know that the ratio of overweight and obesity in Hubei province was controlled smoothly and steadily, with a slight increase in the male overweight group. Since the outbreak of COVID-19, health concerns have become the theme of people’s lives, and physical behavior and heath has been given increasing importance. For the group of overweight and obesity who also failed in the *standard* test, the abilities of agility, explosive, endurance, and muscle strength were needed to be developed accurately as soon as possible. The previous focus of COVID-19 epidemic had targeted on protection of physical health of the global population. However, the influence on mental health, which could be declared as post-coronavirus stress syndrome, would be one of the important consequences of COVID-19 pandemic in the future, and could pose a bigger challenge for global public health (68).

The main strengths of this study are: (1) this study tracked the physical fitness data of the same student population longitudinally for three consecutive years (from 2019 to 2021), in order to assess the impact of COVID-19 crisis on physical fitness among college students in a relative long term; (2) this study contained a large sample of college students from DFC universities/colleges in China with regional information and standardized assessment of physical fitness.

Limitations of our study included reliance on self-report data and lack of field investigation. Findings were based on the Students’ Physical Fitness Monitoring Data Management Center, and the original raw data of physical fitness were measured and uploaded by individual schools. Besides, physical fitness was assessed using the *standard*, so the implications of the findings should be re-considered if generalized to college students from other regions, countries or different ethnicities. Data were collected from the DFC colleges and universities in Hunan province, and it was limited to represent the all country. There were differences in the amount of students among provinces and areas, and the analysis would be partial.

In conclusion, the findings of the present study demonstrate that: (1) students’ physical fitness and health of 10 DFC universities and colleges, both nationwide and in Hubei province, was declined, especially in terms of endurance, which was the most prominent aspect, and the prevalence of overweight and obesity in boys was even worse than that in girls. (2) there was a regional polarization between the ‘excellent/good’ rate and the ‘fail rate, and national coverage of overweight and obesity continued to increase and move southward; the regional distribution of overweight and obesity students from class of 2021st and 2022nd who also failed the standard test showed different characteristics with clustering more in later generation and areas more affected by pandemic. We suggested here: (1) therefore, when formulating physical fitness test standards and promoting physical health for college students, especially students from the western and northeast regions of China, the matching and key interventions should be considered by the authorities; (2) school polices and PE teachers should pay more attention to put training efforts on endurance for all adolescents and strength for boys, and for the group of overweight and obesity who also failed in the standard test, the abilities of agility, explosive, endurance, and muscle strength were needed to be developed accurately as soon as possible; (3) future works is needed to further explore how to maintain and improve physical fitness of college students who will coexist with COVID-19 in the long run.

## Data availability statement

The original contributions presented in the study are included in the article/supplementary material, further inquiries can be directed to the corresponding author.

## Ethics statement

Written informed consent was obtained from the individual(s) for the publication of any potentially identifiable images or data included in this article.

## Author contributions

DC designed the project and edited the manuscript. QJ analyzed the data and wrote the manuscript. XH, ZW, and XD searched the literature on impact of COVID-19 pandemic and lockdown in physical fitness and overweight prevalence of college students. RL edited the manuscript. All authors contributed to the article and approved the submitted version.
